# Impact of the COVID-19 Pandemic on Medical Product Procurement, Prices, and Supply Chain in Zimbabwe: Lessons for Supply Chain Resiliency

**DOI:** 10.9745/GHSP-D-22-00424

**Published:** 2023-10-30

**Authors:** Tatenda T. Yemeke, Farouk A. Umaru, Rashida A. Ferrand, Sachiko Ozawa

**Affiliations:** a Division of Practice Advancement and Clinical Education, UNC Eshelman School of Pharmacy, University of North Carolina, Chapel Hill, NC, USA.; b United States Pharmacopeial Convention, Rockville MD, USA.; c Department of Clinical Research, London School of Hygiene and Tropical Medicine, London, United Kingdom.; d Biomedical Research and Training Institute, Harare, Zimbabwe.; e Department of Maternal and Child Health, UNC Gillings School of Global Public Health, University of North Carolina, Chapel Hill, NC, USA.

## Abstract

**Background:**

The COVID-19 pandemic has disrupted global health supply chains including manufacturing, storage, and delivery of essential medicines, testing kits, personal protective equipment, and laboratory reagents. We sought to document how pandemic impacted the procurement, prices, and supply chain of medical products in Zimbabwe.

**Methods:**

We conducted semistructured in-depth key informant interviews with 36 health system stakeholders in Zimbabwe involved in medicine procurement. Respondents included pharmacists, regulatory officers, and procurement and supply chain management professionals from public and private sectors.

**Results:**

Before the COVID-19 pandemic, respondents described experiencing long-standing resource constraints, medicine shortages, foreign currency shortages, and supply chain inefficiencies. The pandemic exacerbated this situation due to supply constraints, export restrictions, medicine shortages, and movement restrictions that disrupted logistical and stock management systems. Competitive bidding and tendering processes experienced reduced participation by international suppliers. Significant price increases were initially observed among internationally shipped medicines and for personal protective equipment to cover additional freight costs. COVID-19 pandemic impacts were moderated by reduced patient demand and lower health services utilization, resulting in fewer supply shocks and less price volatility. Further, health system adaptations such as switching treatment regimens, modifying dispensing schedules based on stock availability, redistributing stock of medicines among facilities, and new service delivery models such as integrated outreach services helped ensure continued patient access to medicines.

**Conclusions:**

Our findings highlight the need for policies that ensure continuity in access to health services and medical products, even during a pandemic, by avoiding blanket restrictions on medical product exports and imports. Pooled procurement, especially at regional and global levels, with long-term service agreements may help achieve greater resiliency to supply and price shocks from supply chain disruptions. Interventions across manufacturing, trade, and regulatory policy and service delivery models are also needed for supply chain resiliency.

## Abstract (precis)

The authors document how the COVID-19 pandemic disrupted medical product supply chain and procurement in Zimbabwe, impacting medicine access and prices. Policies and interventions are needed to ensure ongoing supply chain resilience.

## Teaser

**Key Findings:**

- Movement restrictions and lockdowns in response to the COVID-19 pandemic affected stock management procedures, procurement processes, and competitive supplier bidding, resulting in price increases and health system disruptions.
- Manufacturing constraints and restrictions of exports from other countries resulted in shortages of medicines and some commodities, supply chain delays, and increased freight costs, which, in turn, significantly increased the cost of certain medical products and impacted access to medicines not related to COVID-19.
- Programmatic and operational shifts within the health system to focus on the COVID-19 pandemic response led to the diversion of human and financial resources from other essential health areas and a decrease in procurement of medicines and commodities that were not related to COVID-19.

**Key Implications:**

- Policymakers should craft policies and interventions to ensure that the supply chain of medical products is resilient for future pandemics and other shocks.
- Policymakers and other health system stakeholders should apply lessons from the COVID-19 supply chain disruptions toward broader health system strengthening efforts.

## Introduction

The COVID-19 pandemic has resulted in more than 600 million cases and nearly 7 million deaths globally as of August 2023.^1^^,^^2^ Beyond the health impacts of COVID-19, there were early concerns about other disruptive impacts on health systems from policy responses and measures adopted to contain the disease. For example, in May 2020, the World Health Organization (WHO) estimated that 80 million children in at least 68 countries were at higher risk of contracting vaccine-preventable diseases due to COVID-19-related disruptions in vaccine delivery, including disruptions of supply chains and vaccine transportation systems and restrictions on travel.^3^^,^^4^ Other WHO reports have documented the pandemic's disruptions of broader essential health services, with many countries reporting reductions in the provision of health services and decreased patient access to health facilities.^5^^–^^7^

Similar concerns have been expressed about the impact of COVID-19 on essential medicines supply chains, with potential for medicine shortages due to supply chain disruptions, limited stockpiling of medicines, and restrictions on medicine exports.^8^^–^^13^ Increased demand and medicine usage related to COVID-19 could also stretch supply chain capacities,^14^ potentially worsening medicine shortages or leading to price increases.^15^ The disruptive impact of COVID-19 on medicine supply chains could be more pronounced in low-and middle-income countries (LMICs), where supply chains and procurement systems already tend to be less resilient.^16^

Available evidence on the impact of COVID-19 on medicine supply chains in LMICs includes a 2021 Global Fund report that concluded that overall, supply risks from COVID-19 impacts were moderate but noted increasing risks to supply from disruptions to freight and logistics.^17^ A multicountry study by the Center for Global Development, focused on COVID-19 impacts in 2021 on supply chains of non-donor-funded essential medicines in LMICs, found evidence of upstream supply disruptions on manufacturing but no evidence of sustained downstream shortages.^18^ Among the few available in-depth country case studies, there is evidence that COVID-19 disruptions impacted availability, prices, and supply chains.^19^^–^^21^ There is a need for more in-depth country case studies, especially among LMICs, that explore the impacts of COVID-19 on medicine supply chains.

We examined the impacts of COVID-19 on procurement, prices, and supply chain of medical products, including essential medicines and other products, in Zimbabwe, a lower middle-income country.^22^ Zimbabwe's health system is based on a primary health care approach. The majority of health facilities are in the public sector, although faith-based and private sector facilities are a significant source of health care in rural and urban areas.^23^ Procurement and financing of medical products is largely donor supported; however, donor funds tend to support specific program areas, mainly HIV/AIDS, TB, malaria, and reproductive maternal, newborn, child, and adolescent health.^24^^,^^25^ Procurement of medical products not supported by donor funding is conducted by the national pharmaceutical company (NatPharm), which also manages the storage and distribution of donor-supported products.^25^ There have been preliminary reports of COVID-19 disruptions to the health system and medicine supply chain in Zimbabwe, including shortages of essential medicines and other products.^21^^,^^26^ This study's findings can help inform policies and interventions in Zimbabwe to mitigate the impacts of supply chain disruptions from COVID-19 and to build resiliency to future supply shocks and can offer lessons for other LMICs with similar contexts. Beyond pandemics, there is a need for ongoing resiliency within the health system and medical product supply chain, and our article synthesizes broader policy implications and lessons from the study findings.

We examined the impacts of COVID-19 on procurement, prices, and supply chain of medical products, including essential medicines and other products, in Zimbabwe.

## Methods

### Study Design

We conducted semistructured in-depth key informant interviews with stakeholders (N=36) across the health system and medicine procurement landscape to gain insights into how COVID-19-related supply chain disruptions impacted procurement and prices of medical products in Zimbabwe and to synthesize broader policy implications from the findings.

### Sampling and Study Procedures

Study respondents were recruited through a combination of purposive sampling and snowball sampling. To ensure representation of the relevant actors involved in procurement, we first drew an initial list of major organizations and institutions involved in procurement of medical products in Zimbabwe based on our understanding of the procurement landscape and then approached key informants from these organizations to participate in the study. The initial key informants then referred additional informants for us to interview based on their roles in procurement and familiarity with interview topics. Respondents in our final sample represented all levels of the supply chain and included those affiliated with procurement organizations that import medical products; the central medicines store, which stores and supplies products to all public health facilities; wholesalers and distributors; national medicines regulatory authority; as well as those providing services through retail pharmacies and health facilities. Participants were drawn from the public and private sectors and collectively represented national-, provincial-, and district-level perspectives. Participants from the public sector included provincial and district pharmacists overseeing medical products in public sector health facilities, while private sector representatives included private retail pharmacists and medical wholesalers and distributors.

Interviews were conducted between April and June 2021 by a trained social scientist with expertise in global health research (TTY) using a hybrid format, with some interviews conducted in person (n=25) and some interviews conducted virtually using Zoom (n=11), based on participant's preferences and logistical considerations. Each interview lasted between 30 minutes and 1 hour, and the interviews were audio-recorded with the respondents' consent. The interviewer took supplemental written notes during each recorded interview. All interviews were conducted in English, and all respondents provided verbal informed consent before participating.

The interviews were conducted using a semistructured interview guide, with follow-up probe questions based on respondents' answers to the initial questions. Interview questions were customized to each participant's organizational sector and role in medicine procurement. For example, respondents located in the upstream of the supply chain were asked to reflect on impacts on the national level, while respondents affiliated with health facilities and retail outlets reflected on downstream impacts. The interview questions elicited respondents' perspectives on whether and how the COVID-19 pandemic impacted procurement and prices of medical products in Zimbabwe. Respondents were also asked to describe how they had adapted their procurement processes in response to disruptions from the pandemic and what impacts on access to medicines they observed.

### Ethical Approval

Ethical approval of the study was sought and obtained from the ethical review boards at the University of North Carolina at Chapel Hill and the Medical Research Council of Zimbabwe. Additional permission to interview key informants affiliated with the Ministry of Health and Child Care was granted by the National Institute of Health Research within the Ministry.

### Data Analysis

Data from key informant interviews were analyzed using thematic analysis in an inductive and iterative manner.^27^^–^^29^ For each interview, we used the audio recordings and interview notes to write qualitative memos that summarized the respondent's background and interview content and highlighted emerging themes. After reading all the memos, we coded them systematically to synthesize the emergent broader themes on the impacts of COVID-19 on the procurement and prices of medical products in Zimbabwe. For example, we applied the code “adaptations to COVID-19” to content that described themes on how respondents and their organizations responded to procurement challenges from the pandemic. We selected representative quotes from key informants' responses that best illustrated their perspectives and the emerging themes across interviews. Further, we used a medicine supply chain diagram to illustrate where in the supply chain various impacts of COVID-19 on procurement and price of medical products were reported. A summary of themes, additional quotes across themes, and the key informant questions are available in the Supplement.

## RESULTS

### Description of Respondents

We interviewed 36 key informants drawn from the Ministry of Health (n=10), donors (n=5), health institutions (n=4), international and local nongovernmental organizations (NGOs) (n=4), medicine wholesalers and distributors (n=3), procurement agencies (n=3), medicine regulatory authorities (n=2), retail pharmacists (n=2), a national medicines advisory committee (n=1), academia (n=1), and an independent consultant (n=1). Collectively, respondents represented perspectives from the national, provincial, and district levels, and their professional roles spanned the entire medicine supply chain from procurement to service delivery points. Respondents' professional roles included provincial and district pharmacists, chief pharmacists of health institutions, regulatory officers, and procurement and supply chain management professionals.

### Summary of Overall Findings

The Figure summarizes the emergent themes from the interviews on the impacts of COVID-19-related disruptions on medical product procurement and supply chain in Zimbabwe and the location of the impacts within the supply chain. Respondents described significant disruptions to medical product procurement and supply chain due to COVID-19 impacts. However, these impacts were moderated by mitigatory factors and adaptations in response to COVID-19. Further, respondents described the disruptions before COVID-19 in the context of the health system in Zimbabwe, with COVID-19 disruption exacerbating existing procurement and health system challenges.

**FIGURE.**
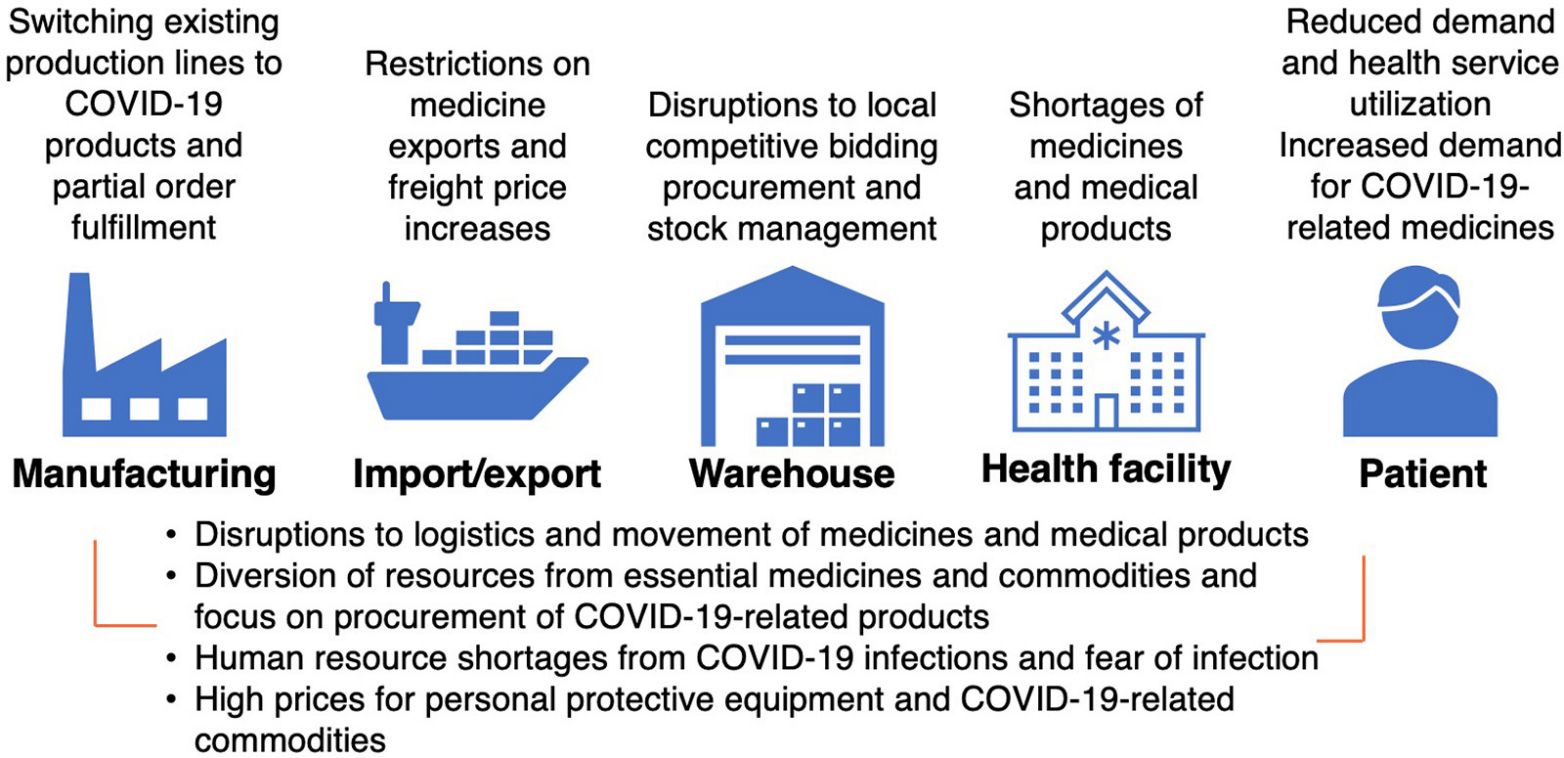
Impact of the COVID-19 Pandemic on Medical Product Procurement and Supply Chain in Zimbabwe

#### Pre-COVID-19 Context

Respondents described long-standing resource constraints, including a lack of credit lines to procure medicines and medical products, and stated that the COVID-19 pandemic exacerbated the situation through diversion of time and resources within the supply chain. A lack of credit lines with medicine suppliers meant that all payments for procurement had to be made up-front, often in a foreign currency that has been perennially in short supply in the country. The local Zimbabwean currency (Zimbabwe dollar) is not easily exchangeable for a foreign currency through formal channels and often depreciates in value; thus, many suppliers prefer payments to be made in foreign currencies. Other challenges that predated COVID-19 include shortages of medicines, especially in the public sector, and supply chain and distribution inefficiencies. For example, respondents reported that there were long-standing shortages of TB prevention medicines that became worse during COVID-19. Zimbabwe's reliance on imports and limited domestic manufacturing capacity for medicines and medical products also made the country vulnerable to supply chain shocks caused by COVID-19.

*I think there were already challenges around the supply chain, around distribution, around the management of NatPharm [national central medical store] and the relationship between NatPharm and the Ministry of Health… Those root issues were there before COVID-19 started, and then COVID-19 exacerbated and if anything, took money, resources, time, and emotion away from those who normally work on other issues in the health sector and forced them to split their time in-between multiple places.* —Respondent, bilateral donor

Respondents described long-standing resource constraints exacerbated by the COVID-19 pandemic through diversion of time and resources within the supply chain.

#### Supply Restrictions From Manufacturing Constraints and Export Restrictions

Respondents reported restrictions in the supply of medicines and medical products attributable to manufacturing constraints. They also described changes and restrictions on exports in important source countries or transit countries. For example, there were shortages of malaria and HIV testing kits after manufacturers had changed their production lines to manufacture COVID-19-related testing kits instead. Consequently, manufacturers faced constraints in meeting demand and rationed quantities of orders that could be fulfilled. For example, there were difficulties in getting orders for laboratory testing cartridges because manufacturers rationed quantities fulfilled.

*It [demand for testing cartridges] got so overwhelming that when countries placed their orders, the manufacturer was the one then determining the amount of product to give to a particular country…That affected our supply chain, the quantity of products that we were receiving from that manufacturer and that really created lots of disruptions, stockouts, in line with the routine testing that we were doing, including also for the COVID-19 testing that we were doing.* —Respondent, Ministry of Health and Child Care

Restrictions on exports of medical products that were implemented by India and South Africa in response to the COVID-19 pandemic resulted in shortages of medicines and supply delays. Both countries are important sources of medicine imports for Zimbabwe, and South Africa is a key transit country where imports pass through its ports. The export restrictions were placed on all medical product categories, including medical products not used for COVID-19 treatment and management. Wholesalers and distributors could still import medicines from South Africa but had to apply for a permit, and obtaining clearances took a long time.

*The South African government stipulated that no drug could be exported out of South Africa unless you have a permit. Whether it be as a source country or a transit country [for the drugs], no drug could leave the South African border without a permit. That created a huge backlog, you can imagine trying to work with SAHPRA [South African Health Products Regulatory Authority] in South Africa trying to get export licenses and permits. It didn't matter whether it was COVID-19 [drugs] or cancer [drugs]…it literally took about 2 months [to obtain permits].* —Respondent, wholesaler/distributor

#### Logistical and Stock Management System Disruptions

Movement restrictions and lockdowns introduced globally in response to COVID-19 resulted in a shortage of freight transport options, which led to increased delivery lead times for imported medicines and medical products. For example, a respondent from a procurement agency described when orders for medicines placed in August 2020 had still not been delivered by May 2021. In-country movement restrictions and COVID-19 infections among staff handling medicines also disrupted local distribution of medical products.

*NatPharm [national central medical store] had been affected also by COVID-19, some of the staff, maybe 50% of the staff got COVID-19 and they shut down for a short period, but when they returned back, they worked in a very precautious way…Also this affected NatPharm's capacity to offload [commodities from transport vehicles].* —Respondent, multilateral donor

Local movement restrictions and diversion of logistic resources, such as vehicles, disrupted stock quantification processes at health facilities needed for the Zimbabwe Assisted Pull System (ZAPS) to adequately procure from the central medicines store. Consequently, quantification of procurement needs relied on inaccurate extrapolations from past consumption, resulting in a mismatch between procurement forecasts and consumption.

*ZAPS is labor-intensive or resource-intensive in the sense that we actually have vehicles going around the different health facilities collecting data. This data is essentially how much they need and for how long. Because our vehicles were now being channeled towards COVID-19 activities, ZAPS itself suffered. I can tell you that in Q2 [second quarter] 2020, Q3 [third quarter] 2020, we could not carry out the ZAPS activities the way we used to.* —Respondent, Ministry of Health

#### Disruptions to Local Procurement Processes

During the COVID-19 pandemic, respondents described a marked reduction in participation in local competitive bidding and tendering processes for medicines and medical products by international bidders and other traditional suppliers. Shortages of active pharmaceutical ingredients, scaling down of business operations due to COVID-19 restrictions, and inability to meet supply timelines were cited as reasons by international and other traditional suppliers for not submitting bids for supply tenders. The reduced number of bidders, especially by more established traditional suppliers, led to reduced competitiveness of the tender process and higher procurement prices.

*Because of COVID-19, we didn't get as many international bidders who participated in our [tendering] process. It actually affected the competitive effectiveness of our procurement system, meaning that you end up getting prices that are higher because of the few people that are participating in your process.* —Respondent, procurement agency

Other barriers to participation by international bidders included an inability to provide sample products for evaluation due to logistical challenges caused by COVID-19 disruptions and bidding processes that required in-person submission and presence during adjudication of bids, which was precluded by COVID-19-related travel restrictions. However, a respondent affiliated with a procurement agency described how COVID-19 provided an opportunity for their organization to transition from a manual procurement and tendering process and adopt an online platform where bidders could participate remotely. Another adaptation reported by respondents included procuring from a short list of suppliers with a good record for quality and performance when a full competitive bidding process was not possible due to COVID-19 restrictions.

Respondents involved in procuring medicines and medical products for public sector facilities described disruptions to funding flows from the central level, as well as foreign currency shortages that led to procurement challenges. This particularly affected public sector facilities' ability to procure from private suppliers, who often had quotations with short validity and were thus sensitive to delays in payment disbursements.

*On funding, we had quite some challenges. The funds [used to] come timely, but it wasn't the case anymore. So, 1 or 2 quarters [3–6 months] were skipped for us to get the funding. Secondly, when you look for quotations from the suppliers, you know procurement is a slow process. But private suppliers, your quotation duration will be 48 hours…If you get a quotation today, they expect you to [take] action within 48 hours, which was quite difficult for government institutions.* —Respondent, Ministry of Health

Respondents described programmatic and operational shifts within the health system to focus on the COVID-19 response at the expense of other functions and reported that this shift led to the diversion of resources from procurement of essential medicines and commodities to procurement of COVID-19-related products. For example, vehicles and human and financial resources were repurposed from other health system areas, including procurement, for the COVID-19 response. Consistent with the health system's programmatic shift toward COVID-19, the procurement agency responsible for procuring medicines for all public sector health facilities experienced a significant decrease in procurement requisitions for medical products that were not related to COVID-19 needs.

*There was sort of a diversion, more focus was on COVID-19 at the expense of other diseases…that's 1 of the challenges that we also faced, that more [procurement] requests were coming from that [COVID-19-related] side at the expense of other requests.* —Respondent, procurement agency

#### Impact on Product Availability and Prices

Respondents described shortages of medicines and medical commodities attributable to COVID-19 disruptions. The classes of medicines most reported as being in short supply included first-line HIV antiretrovirals (e.g., tenofovir, lamivudine, and dolutegravir [TLD]) and preventative TB medicines (e.g., isoniazid and rifapentine), with shortages also being reported for chronic condition medicines, such as antihypertensives. The shortages of HIV antiretrovirals caused delays in long-planned transitions of patients to new TLD-based regimens.

*We were really on a trajectory to get all HIV patients who are eligible to be able to take 3- to 6-month supply of TLD with them home…but because of the shortages, that whole process to the multi-month dispensing has really slowed down and there [were] shortages of TLD across the country. And so, many providers [were] only able to dispense a month at a time.* —Respondent, bilateral donor

Respondents described shortages of medicines and medical commodities attributable to COVID-19 disruptions.

Among nonmedicine products, respondents described shortages of laboratory reagents due to the manufacturing and logistical impacts of COVID-19. Further, the existing stock of reagents intended for other clinical uses was diverted for COVID-19-related use.

*We experienced shortages, specifically with lab reagents, not only the manufacturing, but the delay in freight in getting them into the country and then the diversion of some of those commodities and reagents to COVID-19 testing versus, for example, viral load testing, and so that messed up some of the quantification.* —Respondent, bilateral donor

The impacts of COVID-19 on the prices of medical products in Zimbabwe varied by product type and mode of procurement. There were large increases in the freight cost component of imported products, and some of the increased freight costs were passed along in the price markup. For example, a respondent affiliated with a medicine wholesaler and distributor described freight charges increasing by as much as 500%. Significant price increases were also reported for personal protective equipment (PPE), such as latex gloves and masks, and for other COVID-19-related commodities. Respondents noted that reliance on direct importation of products magnified the effects of freight price increases, while reduced competition due to the reduced number of respondents bidding for supply tenders also resulted in increased unit prices of some products.

*The overall effect is that the unit cost of the product that were bought during the time of COVID-19, especially after March, April, May lockdown, the unit prices became very high because mainly you would be relying on those who had reserved some stock in the country, and they knew that the international world is generally closed. They had little competition. The prices of whatever [goods], either pharmaceutical, lab, medical supplies, and even other things that were used for health, the prices increased significantly.* —Respondent, multilateral donor

According to respondents, there were eventual price reductions, especially for PPE, as more suppliers eventually came on the market and some of the initial global restrictions on travel and logistics in response to COVID-19 eased. Prices of some commodities were reduced to below production cost in some instances due to short shelf life and reduced demand and health service utilization related to COVID-19 disruptions. For example, respondents noted reduced demand for cold and cough medicines as COVID-19 control measures, such as lockdowns, also resulted in reduced incidence of seasonal cold illnesses.

*After the exaggerated increase in prices, there was a subsequent lowering of prices, to even below cost. People were basically doing expiry pricing…The people within even our manufacturing sector, would give you a fresh batch of products for the same price [same price as discounted expiring batches] because, by their prediction, if they didn't do that, they were going to be stuck with an expiring raw material [for which there was no demand].* —Respondent, wholesaler/distributor

Donors providing medical products incurred additional program-level costs associated with emergency shipments and chartering flights for shipments where regular freight routes were not available. However, donor-funded products and commodities did not experience significant price changes, and respondents attributed this to the long-term service agreements and contracts with suppliers that guaranteed a fixed price.

*I didn't see any price volatility… I think largely because for quite a lot of the products there are service level agreements signed by the procurers with suppliers that fixed the price.* —Respondent, international NGO

#### Changes in Demand and Utilization of Health Services

Movement restrictions introduced to contain COVID-19, fear of COVID-19 infection, and health facility closures led to reduced patient access and demand for health services, which in turn impacted the use of medicines and increased expiration of excess stock.

*We had stock [of medicines], we knew the movement in terms of traditional demand…then came COVID-19, the movement of such products was affected. Just as an example, we are now approaching winter, during COVID-19 we are now putting on masks, the children are at home and off school. So, the [medical] conditions that would normally affect us during the winter, it means that they are not there…we saw stagnation in terms of the movement [in the supply chain] of colds and flu remedies, as well as antibiotics.* —Respondent, wholesaler/distributor

Movement restrictions to contain COVID-19, fear of infection, and health facility closures led to reduced patient access and demand for health services, which impacted use of medicines and increased expiration of excess stock.

Simultaneously, respondents also reported increased demand for COVID-19-related medicines, including unregistered and unapproved medicines. A participant affiliated with a national medicines advisory committee also described political pressure on the committee to endorse medicines that were not scientifically proven as COVID-19 therapies, such as hydroxychloroquine and ivermectin.^30^^,^^31^

*Then there is demand now for products for the treatment of COVID-19, which traditionally we didn't have. For example, initially, hydroxychloroquine, we had stock, we had registered it for the treatment of lupus and arthritis, so we had few stocks just for the treatment of those conditions and then suddenly there is an upsurge [demand for COVID-19 treatment].* —Respondent, wholesaler/distributor

#### Mitigatory Factors

Respondents described some factors that mitigated the impacts of COVID-19-related disruptions on the supply chain of medicines and medical commodities in Zimbabwe. Donor-funded commodities, such as HIV and maternal and child health products, were perceived to be resilient to supply shocks and price volatility. Global pooled procurement of these products was identified by respondents as being key to this resiliency compared to products that were procured at the national level, where there are no equivalent long-term supply agreements specifying volumes and prices. Zimbabwe's minimum/maximum stocking system, where health facilities have buffer stock available, was also cited as key to smoothing out the effects of COVID-19-related supply shocks.

*In terms of stock rupture…I think that was limited…it could have been worse… because the country works on minimum and maximum stock levels of what stock sit at the facilities and what stock sits at the central level…and that stock is about 9 months. So, for your medicines that are required for chronic conditions, which is where it's more predictable, the forecast for chronic conditions is more stable and less volatile. So, I guess the ruptures [stock shortages] were less extreme because of those technical considerations.* —Respondent, international NGO

Another important mitigatory factor was the availability of funding to support the procurement of commodities through a multilateral donor-funded Health Development Fund (HDF), as well as existing reserve stocks of essential medicines that had been procured under the HDF before COVID-19. A respondent who oversaw a multicountry health sector portfolio for a multilateral donor stated that other countries in the region that did not have support programs like the HDF had not had the same commodity resiliency as Zimbabwe. However, the respondent also noted that the COVID-19 pandemic had also disrupted and delayed the process for a new phase of the HDF financing mechanism, hence, potentially impacting the continuity of a key source of resiliency.

Zimbabwe's recent experience with environmental shocks from Cyclone Idai in 2019 was also cited as an important mitigatory factor. In response to disruptions from Cyclone Idai, donors had mobilized medicines and resources. Leftovers of those resources and medicines were still available in the country when the COVID-19 pandemic began, which helped ease supply disruptions.

#### Health System Adaptations to COVID-19

Respondents described several adaptations made by the health system that helped mitigate the impacts of COVID-19 on the procurement and supply chain of medicines and medical commodities. Clinicians switched treatment regimens and modified dispensing schedules based on stock availability. For example, patients were switched to alternative regimens when there was a shortage of some types of antiretrovirals. Where stock was available, facilities introduced multi-month dispensing of medicines for chronic health conditions to ensure continued access amid challenges patients faced in reaching health facilities. New service delivery models, such as integrated outreach services, online ordering, and home delivery of medicines, were other key innovations used to ensure continued medicines access during the COVID-19 pandemic. Emergency ordering from the central medical store was used to ensure that facilities had the minimum required stock levels and to redistribute stocks among facilities. Other adaptations included repurposing existing laboratory testing products for other disease diagnostics for the COVID-19 testing program.

*We had to engage in a robust program of product redistribution so that the little commodities that were already available in country could at least be pinched to last more than we had anticipated.* —Respondent, Ministry of Health

Another reported adaptation was increased use of special medicine imports that were exempt from registration requirements under section 75 of medicine regulatory rules.^32^ Respondents affiliated with wholesalers and distributors described using this flexibility to import medicines and products that were in short supply on the market.

*There was a spike in Section 75 applications, which are basically special imports for unregistered medicines. Even from my experience with our community pharmacies, there was, I think, over 350% increase in special imports. Because something is not available on the market, we could get authorization to import it directly and get it to the market…it could be specific patient-driven Section 75s or the bulk Section 75s for medicines that were not available.* —Respondent, wholesaler/distributor

## DISCUSSION

Our study found that the COVID-19 pandemic disrupted the supply chain of medical products in Zimbabwe, leading to price increases and shortages of some commodities, as well as increased freight and logistics costs. These impacts were exacerbated by a pre-COVID-19 context in which there were already long-standing challenges, including shortages of medicines and supply chain inefficiencies. However, the impacts of COVID-19 were moderated by several mitigatory factors and health system adaptations to cope with the disruptions from COVID-19 that resulted in some resiliency to the supply shocks. Further, there were eventual price reductions as supplies improved and trade restrictions eased, while prices of donor-funded products did not experience any significant price changes. This study adds to the growing literature on the impacts of COVID-19 on medical product supply chains but is among the first to employ in-depth qualitative methods in Sub-Saharan Africa.^17^^–^^21^

### Policy Implications and Lessons for Health System and Supply Chain Resiliency

The Table presents our key findings alongside policy implications and lessons to strengthen resiliency in supply chains. We found that donor-funded medical products, using global pooled procurement with long-term supply agreements, were more resilient to supply and price shocks from COVID-19 supply chain disruptions. This suggests that pooled procurement, especially at regional and global levels, may be key to building resiliency and minimizing impacts on medicine supply and prices.^33^^–^^35^ Thus, Zimbabwe and other LMICs should consider greater use of pooled procurement of medical products as a strategy to mitigate the impacts of future pandemics and other supply shocks. Examples of such existing regional pooled procurement initiatives include the Pan American Health Organization Strategic Fund, a pooled procurement for the Organization of Eastern Caribbean States, and pooled procurement initiatives under the South African Development Community.^36^ Beyond pooled procurement at regional or global levels, pooled procurement at national and subnational levels has also been shown to improve procurement outcomes and could be an important interim step to ensuring supply chain resiliency.^37^^,^^38^ However, establishing effective pooled procurement mechanisms at regional levels has been shown to be challenging due to the need to align the goals of actors with diverse characteristics.^39^^,^^40^ At the subnational level, establishing pooled procurement mechanisms among similar organizations, such as faith-based organizations that are an important component of the health system in Zimbabwe, might be more attainable in the short term.^41^

**TABLE.** Policy Implications and Lessons From Findings on the Impact of the COVID-19 Pandemic on Medical Product Procurement and Supply Chain in Zimbabwe

Policy Domain	Findings	Policy Implications/Lessons
Manufacturing	Reliance on imports and limited domestic manufacturing caused vulnerability to supply chain shocks, including initial high prices for products such as personal protective equipment. Increased domestic production eventually alleviated shortages and helped reduce prices.	Policies are needed to increase and sustain domestic production of medical products to mitigate supply shocks from disruptions to international supply chains.
Trade policy	Broad restrictions in trade and exports of medical products caused shortages and supply chain delays.	Bilateral and multilateral conventions are needed to prevent blanket restrictions on the movement of medical products during emergencies. Broader trade policies that prioritize access to essential medicines and medical products are essential for public health.
Procurement	Competitive bidding processes that required in-person submission of bids and presence during the adjudication of bids precluded participation by international bidders who faced travel restrictions. Products procured under global pooled procurement with long-term supply agreements were more resilient to supply and price shocks.	Modernizing procurement processes by adopting technological solutions, such as online-based bidding platforms, can build resiliency and flexibility and help sustain the competitiveness of bidding processes. Pooled procurement at national, regional, and global levels could be an important strategy for fostering supply chain resiliency and improving procurement outcomes.
Health system financing	Disruptions to funding flows from the central level to health facilities hampered health facilities' ability to procure commodities. The availability of a multilateral support fund for the health sector ensured there were resources to support commodity procurement. Reserve stocks of essential medicines purchased under the fund were available to bridge supply constraints.	Health system financing reform, including decentralizing funding to local levels, could empower health facilities to improve procurement planning without funding flow uncertainties. Coordination of development assistance for the health sector among partners and long-term strategic funding investments can foster broader health system resilience.
Regulatory policy	Provisions for waiver of registration requirements in the regulatory framework allowed imports of products that were in short supply locally.	Regulatory frameworks should have flexible provisions to allow market needs to be met during times of constrained supply and where products are not available locally. There is a need to ensure that products imported under special provisions are also quality assured.
Service delivery models	Innovative service delivery models, such as integrated outreach services, helped ensure continued access to health services amidst access restrictions.	Health systems should explore innovative service delivery models that promote access to health services, encompassing hard-to-reach populations.

Zimbabwe and other LMICs should consider greater use of pooled procurement of medical products as a strategy to mitigate the impacts of future pandemics and other supply shocks.

The impact of restrictions on exports and the movement of medical products, which resulted in shortages and supply chain delays, highlights an urgent need for policymakers to ensure continuity in access to medical products during pandemics and other similar emergencies.^42^ Respondents reported that these restrictions were of a blanket nature and impacted classes of medical products that were not related to COVID-19, such as pediatric cancer medicines. Thus, the restrictions likely led to downstream consequences, including increased mortality and morbidity from lack of timely access to essential medical products. Such effects would have also been compounded by overall decreases in demand and access to health services due to COVID-19 that were observed in Zimbabwe and other LMICs.^5^^–^^7^ Future studies should investigate the public health impacts associated with supply chain disruptions, restrictions on medical product access, and reduced service utilization due to COVID-19, such as impacts on routine immunization uptake or on HIV treatment.^43^^–^^46^

The disruptions to the local procurement systems noted by respondents underscore the need to improve the structure and modify procurement procedures to promote resiliency in the system. For example, some of the barriers to participation in tenders by international bidders and other traditional suppliers during the pandemic included requirements to submit physical copies of bids and being present in person at adjudication, which was not feasible due to pandemic-related movement restrictions. Adopting the use of online bidding platforms could allow greater participation by bidders and help preserve the competitiveness of the procurement system. Online platforms and other information technology interventions have been adopted by the medicine regulation context in Zimbabwe in response to COVID-19 impacts and can offer lessons for the procurement context.^47^ When possible, policymakers should consider developing policies to promote and increase domestic production of medical products to reduce reliance on imports that are vulnerable to logistical and supply chain disruptions. For example, respondents in our study noted that Zimbabwe's reliance on imports made it vulnerable to supply chain disruptions during the pandemic. Zimbabwe has traditionally had a strong pharmaceutical product manufacturing base compared to regional peers, with an estimated 14 manufacturers who are compliant with good manufacturing practices; however, recent economic challenges have decimated the output of local manufacturers.^40^^,^^48^^,^^49^ Notwithstanding these challenges, increased domestic production of PPE was credited with increased supply and eventual reduction in unit costs of PPE products.

Our study also identified several mitigatory factors and adaptations that were made to moderate the supply chain disruptions from the pandemic, which could offer potential lessons for other LMICs. Key among these factors was the ready availability of funds through the HDF to support the procurement of medical products, which suggests the need for countries to plan contingency financing to support procurement during emergencies.^50^^,^^51^ However, such financing would need to be holistic and consider the whole health system, as evidenced by the diversion of resources from essential medicines procurement toward the COVID-19 response. Further, having legislative and regulatory frameworks in place, such as the special waiver provision for importation of medicines not registered on the local market, likely enabled swift response by wholesalers to meet gaps in medical product supply and forestall some shortages. Therefore, medicine regulators should consider adopting and expanding similar legal provisions that enable supply chain actors to meet market demand for medical products during times of strained supply.

Medicine regulators should consider adopting and expanding legal provisions that enable supply chain actors to meet market demand for medical products during times of strained supply.

### Limitations

There are some limitations to our study. The COVID-19 pandemic is still ongoing, and there might be a time lag in how the pandemic affects the supply chain. The cross-sectional nature of our study means that we may have missed some longitudinal effects of the pandemic. For example, respondents noted that Zimbabwe had some buffer stocks of medicines that had been procured in response to a tropical cyclone disaster, and some of those medicines, along with other reserves, may have helped smooth out earlier supply shocks. Moreover, the reduction in demand and utilization of health services due to movement restrictions noted in our study, including the closure of outpatient services at major facilities, could have masked the effects of the supply shocks. Furthermore, in Zimbabwe, before COVID-19, there were existing medical product supply chain issues including essential medicines shortages, which might have made it difficult to parse out COVID-19-specific effects. While this study interviewed 36 respondents involved in health care and medicine procurement, we may not have captured the COVID-19 supply chain impacts in all aspects across medicines and subpopulations. Future studies should consider using quantitative procurement and price data to corroborate and triangulate with the qualitative findings of our study, as well as explore temporal trends in the impacts as the COVID-19 pandemic continues to evolve.

## CONCLUSION

We found that the COVID-19 pandemic disrupted the supply chain, which impacted the procurement and prices of medical products in Zimbabwe. Our findings highlight the need for policymakers to craft policies and interventions to ensure that the supply chain of medical products is resilient for future pandemics and other shocks. Movement restrictions and restrictive trade policies on medical products should be weighed carefully against the need to ensure continued access to health services and essential medical products. Pooled procurement, especially at regional and global levels, may achieve greater resiliency to supply and price shocks from global pandemic disruptions.

## Supplementary Material

GHSP-D-22-00424-supplement.pdf

## Notes

**Peer Reviewed**

**First published online:** September 8, 2023.
