# Supplementary material for: Impact of the COVID-19 Pandemic on Medical Product Procurement, Prices, and Supply Chain in Zimbabwe: Lessons for Supply Chain Resiliency
Source: Glob Health Sci Pract. 2023 Oct 30;11(5):e2200424. doi: 10.9745/GHSP-D-22-00424 (PMC10615236; doi:10.9745/GHSP-D-22-00424)
Supplement: GHSP-D-22-00424-supplement.pdf [file GHSP-D-22-00424-supplement.pdf]

Supplement to: Yemeke TT, Umaru F, Ferrand RA, Ozawa S. Impact of COVID-19 on procurement, prices, and supply chain of medical products in Zimbabwe: lessons for supply chain resiliency. *Glob Health Sci Pract*. 2023;11(5):e2200424. https://doi.org/10.9745/GHSP-D-22-00424

Impact of COVID-19 on Procurement, Prices, and Supply Chain of Medical Products in Zimbabwe: Lessons for Supply Chain Resiliency

Supplemental Materials

Table of Contents

Summary of themes from key informant interviews 2
Additional representative quotes across themes 5
Questions for key informants 7

Supplement to: Yemeke TT, Umaru F, Ferrand RA, Ozawa S. Impact of COVID-19 on procurement, prices, and supply chain of medical products in Zimbabwe: lessons for supply chain resiliency. *Glob Health Sci Pract*. 2023;11(5):e2200424. https://doi.org/10.9745/GHSP-D-22-00424

Summary of themes from key informant interviews

Theme	Summary
Pre-COVID-19 context	• Long-standing resource constraints and lack of credit lines<br>• Pre-existing shortages of medicines, especially in public sector, and inefficient supply chain and distribution system<br>• Reliance on imports and lack of domestic manufacturing made country vulnerable to shocks
Supply restrictions from manufacturing constraints and export restrictions	• Manufacturers switched production lines to more lucrative COVID-19 testing products instead of Malaria/HIV testing kits<br>• Manufacturers rationed orders that could be fulfilled and cut access to some medical products<br>• Important source countries, such as South Africa and India, imposed restrictions on medicine exports, causing shortages and delays in imports
Logistical and stock management system disruptions	• Challenges in securing freight transport for imported commodities led to increased delivery lead times<br>• Movement restrictions in the country and COVID-19 infections among workers disrupted in-country distribution and logistics<br>• Movement restrictions and diversion of resources to COVID-19 response disrupted the stock quantification process under the assisted pull procurement system from the central medical store and led to reliance on inaccurate demand forecasts<br>• Inaccuracies in extrapolated quantifications and mismatch between procurement forecast and consumption led to expirations, shortages, and storage issues
Disruptions to local procurement processes	• Disruptions to competitive bidding and tendering processes, with reduced participation by international bidders and other traditional suppliers<br>• Disruptions to funding flows from central level to public sector facilities made it harder to procure from private suppliers<br>• Scarcity of foreign currency led to 'middle-men' providing funds for procurement at a markup<br>• Diversion of resources (vehicles, human, and financial) from essential medicines and commodities to focus on procurement of COVID-19 related products

Supplement to: Yemeke TT, Umaru F, Ferrand RA, Ozawa S. Impact of COVID-19 on procurement, prices, and supply chain of medical products in Zimbabwe: lessons for supply chain resiliency. *Glob Health Sci Pract*. 2023;11(5):e2200424. https://doi.org/10.9745/GHSP-D-22-00424

Theme	Summary
Impact on product availability and prices	• Shortages of antiretroviral drugs, TB, anesthetics, chronic condition medicines<br>• Shortages of HIV and malaria testing kits and lab reagents<br>• Significant increases in prices of freight for imports<br>• Significant price increases and high prices for personal protective equipment and COVID-19 related commodities; however, prices eventually reduced with improved supply and more competition<br>• Reliance on direct importation and reduced number of respondents in competitive bidding led to higher prices for other non-COVID-19 related commodities<br>• Prices of some commodities were reduced, to below cost in some instances, due to short shelf life and reduced demand and health service utilization<br>• No price changes for donor funded commodities bought through global pooled procurement under long-term supply agreements
Changes in demand and utilization of health services	• Movement restrictions and fear of infection led to reduced patient access and utilization of health services<br>• Increased demand for COVID-19 related medicines, including unregistered/unapproved medicines<br>• Decreased demand for non-COVID-19 medicines due to low health service utilization and reduced incidence of seasonal illnesses, leading to increased expirations
Mitigatory factors	• Donor-funded commodities using global pooled procurement were resilient to supply shocks and price changes<br>• Stock reserves and buffers were available due to ‘minimum/maximum’ stocking system<br>• Availability of financing from multilateral donor-funded Health Development Fund to support commodity procurement

Supplement to: Yemeke TT, Umaru F, Ferrand RA, Ozawa S. Impact of COVID-19 on procurement, prices, and supply chain of medical products in Zimbabwe: lessons for supply chain resiliency. *Glob Health Sci Pract*. 2023;11(5):e2200424. https://doi.org/10.9745/GHSP-D-22-00424

Theme	Summary
Health system adaptations to COVID-19	• Switching of treatment regimens and modified dispensing schedule based on availability<br>• Redistribution of stocks among facilities, multi-month dispensing for chronic conditions, and emergency ordering for facilities below minimum stock levels<br>• Waivers for direct importation of medicines and commodities not registered in Zimbabwe<br>• Repurposing of laboratory testing products from other disease areas towards COVID-19 testing<br>• New service delivery models, such as integrated outreach, online ordering, and home deliveries to ensure continued access to medicines<br>• Adopting online-based platforms for tendering and bidding processes<br>• Using shortlists of suppliers with history of performance and quality when a full competitive bidding process was not possible<br>• Lobbying for increased domestic production

Supplement to: Yemeke TT, Umaru F, Ferrand RA, Ozawa S. Impact of COVID-19 on procurement, prices, and supply chain of medical products in Zimbabwe: lessons for supply chain resiliency. *Glob Health Sci Pract*. 2023;11(5):e2200424. https://doi.org/10.9745/GHSP-D-22-00424

Additional representative quotes across themes

Theme	Representative quotes
Pre-COVID-19 context	"We have always faced a problem with TB preventative treatment medicines, we are talking of Isoniazid, Pyridoxine, 3HP… those medicines have always had an irregular supply but with this whole situation [Covid-19 pandemic] things just got out of hand and it became worse" – [Foreign government technical agency]
Supply restrictions from manufacturing constraints and export restrictions	"For some of the HIV test kits, there was a complete stop because the production lines had been changed to COVID-19. For malaria as well, there was a significant reduction in terms of production lines for malaria test kits… so there now had to be rationalization and prioritization… so that led to shortages of those test kits" – [International implementing organization]<br><br>"A lot our product does come from South Africa because of our proximity to South Africa. A lot of these companies have distribution hub in South Africa, and they distribute from there to their agents in SADC [Southern Africa region]. A lot of the big multinationals have hubs in Johannesburg where they ship from…So that [restrictions on exports from South Africa] created a significant non-availability in the market and that's across the board, it didn't matter what disease you had." – [Wholesaler/Distributor]
Logistical and stock management system disruptions	"It was really a challenge [increased delivery lead times], even up to now, there is one scenario whereby we placed orders around August [2020] and up to now [May 2021] those goods haven't yet been delivered completely. The challenge is shipping, supply-chain disruption." – [Procurement agency]<br><br>"There is a lot of expiries that have happened this season [pandemic], because of using forecast values based on historical events. Because this [pandemic] was a change, there are some products for which the forecast was wrong, because of breaks in the movement of people, breaks in movement of other infections [reduction in incidence of other infectious diseases]." – [Government ministry]
Disruptions to local procurement processes	"Restrictions of the lockdown contributed also to some of the human capital disruptions…There was disruption in how our

Supplement to: Yemeke TT, Umaru F, Ferrand RA, Ozawa S. Impact of COVID-19 on procurement, prices, and supply chain of medical products in Zimbabwe: lessons for supply chain resiliency. *Glob Health Sci Pract*. 2023;11(5):e2200424. https://doi.org/10.9745/GHSP-D-22-00424

Theme	Representative quotes
	human resource capital would contribute to the normal flow of things. Placing of orders was then delayed, collection of order and distribution of medicines was then delayed et cetera." – [Government ministry]<br>"The challenge when COVID-19 came onboard was because suddenly the politicians were meeting their colleagues somewhere and people would say 'You can use Hydroxychloroquine, you can use Interferon, you can use this'. We were expected to endorse some of these medicines where we felt that there was not enough scientific for their use. For me, it was fairly straightforward, if you don't have scientific evidence then you don't include it." – [National medicines advisory committee]
Impact on product availability and prices	"The freight charges were very high. We had seen a rise in freight costs from a percentage of about 5% to even up to a high of more than 30% to 35%...I also talk about medical supplies, not pharmaceutical alone…at some point the freight costs for importing latex gloves was almost more than the cost of the latex gloves themselves." – [Multilateral donor]<br><br>"We saw the prices shooting up…In many instances we do a tender, and we find that prices are too high [than] what we expect. We actually decided not to award the tender and we started over again [invitation for bids]" – [Health Institution]
Mitigatory factors	"We were affected because some of the manufacturers had closed, like in India due to the initial lockdown. So, you find that things like Paracetamol and so forth, there were delays in terms of coming into Zimbabwe. But the actual impact was not felt much because we had adequate stock to cover for at least the next 3 months." – [Multilateral donor]

Supplement to: Yemeke TT, Umaru F, Ferrand RA, Ozawa S. Impact of COVID-19 on procurement, prices, and supply chain of medical products in Zimbabwe: lessons for supply chain resiliency. *Glob Health Sci Pract*. 2023;11(5):e2200424. https://doi.org/10.9745/GHSP-D-22-00424

Questions for key informants

1. How did COVID-19 affect procurement of essential medicines, including vaccines and contraceptives, in Zimbabwe?
2. What procurement-related challenges did/have you encountered as a result of COVID-19?
3. Have you had to change procurement processes as a result of COVID-19? If so, how?
4. How have prices of essential medicines been affected as a result of COVID-19?
5. How did COVID-19 impact demand for medicines?
6. How did COVID-19 impact patient access to medicines?
7. What have been the socio-economic effects (if any) of COVID-19 on end users of medicines?
8. Are there publicly available data on procurement activities that you could share that would help me understand the impact of COVID-19 on procurement in Zimbabwe?
9. Are there other informants I should interview?
